# Wastewater Treatment Using Membrane Bioreactor Technologies: Removal of Phenolic Contaminants from Oil and Coal Refineries and Pharmaceutical Industries

**DOI:** 10.3390/polym16030443

**Published:** 2024-02-05

**Authors:** Mohd Jahir Khan, Agung Wibowo, Zoheb Karim, Pattaraporn Posoknistakul, Babasaheb M. Matsagar, Kevin C.-W. Wu, Chularat Sakdaronnarong

**Affiliations:** 1 Department of Chemical Engineering, Faculty of Engineering, Mahidol University, 25/25 Putthamonthon 4 Road, Salaya, Putthamonthon, Nakhon Pathom 73170, Thailand; mohammad.jah@mahidol.ac.th (M.J.K.); agung.wib@student.mahidol.ac.th (A.W.); pattaraporn.pos@mahidol.edu (P.P.); 2 MoRe Research Örnsköldsvik AB, SE-89122 Örnsköldsvik, Sweden; zoheb.karim@alfalaval.com; 3 Department of Chemical Engineering, National Taiwan University, Taipei 10617, Taiwan; matsagar03@ntu.edu.tw; 4 Department of Chemical Engineering and Materials Science, Yuan Ze University, Chung-Li, Taoyuan 32003, Taiwan

**Keywords:** membrane bioreactor, phenolics, industrial effluents, contaminants removal, water pollution, wastewater treatment, human health, sustainability

## Abstract

Huge amounts of noxious chemicals from coal and petrochemical refineries and pharmaceutical industries are released into water bodies. These chemicals are highly toxic and cause adverse effects on both aquatic and terrestrial life. The removal of hazardous contaminants from industrial effluents is expensive and environmentally driven. The majority of the technologies applied nowadays for the removal of phenols and other contaminants are based on physio-chemical processes such as solvent extraction, chemical precipitation, and adsorption. The removal efficiency of toxic chemicals, especially phenols, is low with these technologies when the concentrations are very low. Furthermore, the major drawbacks of these technologies are the high operation costs and inadequate selectivity. To overcome these limitations, researchers are applying biological and membrane technologies together, which are gaining more attention because of their ease of use, high selectivity, and effectiveness. In the present review, the microbial degradation of phenolics in combination with intensified membrane bioreactors (MBRs) has been discussed. Important factors, including the origin and mode of phenols’ biodegradation as well as the characteristics of the membrane bioreactors for the optimal removal of phenolic contaminants from industrial effluents are considered. The modifications of MBRs for the removal of phenols from various wastewater sources have also been addressed in this review article. The economic analysis on the cost and benefits of MBR technology compared with conventional wastewater treatments is discussed extensively.

## 1. Introduction

Expansive industrialization and human activities have led to a huge increase in hazardous chemicals in water bodies, causing massive water pollution [1]. Industries are the major cause of pollution in all ecosystems since large amounts of toxic chemicals are released either directly or indirectly into water bodies. It is believed that approximately 220 billion tons of chemicals from all sources are released annually [2]. Wastewater discharged from different industries is hazardous to terrestrial and aquatic life, since it changes the physical, chemical, and biological properties of receiving water bodies [3,4]. Phenol is a vital part of many products, including paints, cosmetics, medicines, and lubricants. It is used as an intermediate in the production of phenolic resins, especially in the phenol-formaldehyde resins which account for more than 35% of overall uses. Furthermore, plastic precursors such as polycarbonates and epoxide resins are produced by the condensation of phenol with acetone to produce bisphenol-A. Cyclohexanone, an important component used in the manufacturing of nylon, is another significant chemical that can be made from phenol. Other applications of phenol and its derivatives comprise their role as flexible precursors for the manufacturing of pharmaceuticals like aspirin and pharyngitis medicines, carbolic soap; cosmetics like sunscreen, hair dye, and skin lightening products; and aviation industry like a component of industrial paint strippers [5,6]. As a result, phenol and its derivatives are found in the effluents of different industries including petrochemical, fiberglass, textiles, and coking industries. The concentrations of phenols in the wastewater discharged from different industries are varied such as refineries (6–500 mg L^−1^), coking operations (28–3900 mg L^−1^), coal processing (9–6800 mg L^−1^), and petrochemical plants (2.8–1220 mg L^−1^). The concentration of phenols from pharmaceuticals, plastics, wood products, paint, pulp, and paper industries are about 0.1–1600 mg L^−1^ [7,8,9]. For the sake of protecting human health and the ecosystem, the removal of phenol from wastewater is necessary. These circumstances are driving the scientific community to find effective techniques for environmental cleanup. The most effective strategies in protecting the environment are reducing the generation of toxic chemicals, implementing advanced treatment technologies, and improving wastewater management. It is necessary to use innovative methods to enhance hydrological cycle management in the public and industrial sectors to prevent water pollution and environmental changes. Applying efficient and cutting-edge methods can enable industrial wastewater to be recycled for uses in agriculture and industries [10,11,12].

A traditional wastewater treatment entails many processes for pollutants’ removal before their release into the environment. In these technologies, several steps are involved depending on the region, regulation & policy and level of treatment needed. The treatment of phenolic compounds is extremely challenging for the above-mentioned industrial effluents. The common methods applied by chemical industries are based on activated sludge digestion, solvent extraction, chemical treatment, and adsorption [13,14]. Several state-of-the-art methods based on oxidation processes have been thoroughly investigated to remove phenolic compounds completely. This group of methods includes chemical, photochemical, and electrochemical oxidation [15,16]. In this context, many sophisticated procedures based on membrane separation methods like reverse osmosis and micro/ultrafiltration have also been considered. To remove various organic contaminants from effluents, membrane-based technologies have extensively been studied for the removal of phenolic compounds. The main benefits of these technologies are their small footprint and high effluent quality. However, membranes’ short lifetime due to fouling caused by particles and colloids in the feed stream and high energy consumption are the major limitations of these technologies [17,18].

An important technology called membrane biological reactors or membrane bioreactors (MBRs), which combines biological processes with membrane filtration, is an effective alternative to traditional wastewater treatments. In MBRs, membrane filtration is used to separate microbes and substances that has been degraded in the reactor by microorganisms. The MBR technology has shown significant progress over the past few years because of its high potential in producing quality effluent due to the significantly improved membrane efficiency and selectivity with lower cost, and thus MBR is now regarded as a matured technology for wastewater treatment [19,20]. The MBR improves biological activity management, leads to pathogen-free effluents, reduces plant water footprint, and is operated effectively for high organic loading rates [21]. Treatments of phenolic compounds are interesting areas where MBR technology has been extensively applied, but it still requires attention for a detailed experimental and economical evaluation. The construction of versatile bioreactors that can be included in different treatment processes is required to remove harmful compounds and makes the treated water reusable.

This article provides a detailed literature study of phenol degradation and removal using membrane biological reactor technologies. The degradation of phenols and their derivatives in both aerobic and anaerobic conditions by bacteria, algae, and fungi is well summarized. Furthermore, the capital expenditures (CAPEX) and the operational expenses (OPEX) of conventional and modified MBR technologies compared with a traditional activated sludge treatment on phenol degradation are thoroughly investigated. Additionally, the advantages of biobased cellulose membranes in MBRs are also discussed. The biobased cellulose membrane separation might become a next-generation approach toward a circular economy. We believe that this review article not only provides recent and in-depth information on membrane bioreactor technology but is also helpful in searching for novel microorganisms that exhibit high efficiency in phenol degradation. This work provides researchers with new insights that are beneficial to design new ways for phenols degradation and removal from industrial wastewater.

## 2. Phenols and Their Derivatives Posing a Human Health Risk

Phenols are highly toxic, even at low concentration, because of their hydrophilic and hydrophobic properties. They are one of the most toxic organic contaminants in wastewater causing necrosis and protein denaturation [22]. Phenols are non-biodegradable, so they remain in the ecosystem for a long time. They are soluble in water, oil, and most organic solvents, including alcohols, ethers, and ketones [23]. Phenol and its methyl derivatives were recognized as stable, priority chemical pollutants by the US Environmental Protection Agency (EPA) in 1979 [24]. They burn the skin and eyes as they are quickly absorbed. According to Alshabib and Onaizi’s report, acute phenol exposure can cause complication of the central nervous system and leads to collapse and loss of consciousness. Exposure to phenol fume can additionally cause lung edema, coughing, dyspnea, cyanosis [25], and severe damage to internal organs including kidney, liver, spleen, heart, and lung. Biochemical system failure, infertility, and neuropsychiatric disorders are also associated with acute phenol poisoning [26]. According to the Occupational Safety and Health Administration (OSHA) and the American Conference of Governmental Industrial Hygienists (ACGIH), the maximum limit of phenols that can come into contact with the skin is less than 5 mg L^−1^. It is well known that the ingestion of 1 g of phenol is fatal for humans. Apart from human beings, phenol causes damage to aquatic life when the concentration is more than 1 mg L^−1^. A strict effluent discharge limit of less than 0.5 mg L^−1^ is, therefore, applied. The maximum permitted quantity of phenol in non-chlorinated water, as adapted from several water supply regulations, is 0.1 mg L^−1^ (100 ppb), while that in chlorinated water is in a range of 0.001–0.002 mg L^−1^ (1–2 ppb) [25,27,28].

Phenols and their derivatives are directly linked to the dyeing process. Since many dyes are generated from known carcinogens, concerns over their possible toxicity and carcinogenicity have led to research on the toxic and hazardous potential of dyes and their intermediates. The fused aromatic structure of anthraquinone-based dyes such as solvent green 3, solvent blue 35, dispersed red 9, and benzanthrone makes them the most resistant to oxidation [29,30,31]. Due to their high color intensity and brilliance, basic dyes are more challenging to decolorize [32]. Complex metal dyes, like those made of chromium, are carcinogenic. Most basic dyes are poisonous and many of them are recognized as possible carcinogens; therefore, they could pose a risk to human health [33,34].

The reduction of phenols in industrial effluents is of great environmental concern due to their complex chemical content, high toxicity to aquatic and terrestrial life, and low biodegradability. The toxicity of phenols and other contaminants in various industrial effluents should be monitored before they are discharged into water bodies. There are several assays by which the toxicity of phenols is monitored. By using aquatic species in bioassays one can assess the toxicity of phenols. The toxicity of pre- and post-treated textile wastewater was evaluated by Castro et al. with four different organisms, viz., *Aliivibrio fischeri*, *Raphidocelis subcapitata*, *Daphnia magna*, and *Lemna minor*. These organisms represent different trophic levels. The study showed that the untreated effluent was very toxic, with *A. fischeri* being the most sensitive organism. While the toxicity of the effluent after the treatment was significantly reduced for *A. fischeri*, *R. subcapitata*, and *L. minor*, the treated effluent was still toxic for *D. magna* [35]. Besides from the above-mentioned study, the toxicity of synthetic phenol has been examined with different groups of aquatic organisms, including bacterial culture, the algae *Scenedesmus quadricauda*, the crustacean *Daphnia pulex*, and the fish *Oncorhynchus mykiss.* The report demonstrated that phenol was more toxic to fish, followed by crustaceans and then green algae. Among all aquatic organisms tested, the bacterial culture was the least sensitive to phenol toxicity [36]. Kahru et al. studied the toxicity of eight phenolic compounds present in oil shale industry wastewaters. The organisms used for testing represent different trophic levels, such as photobacteria, protozoa, crustacean, and microalgae. The wastewater contained five monobasic phenolics—phenol, *p*-cresol, 2,4-dimethylphenol, 2,3-dimethylphenol, and 3,4-dimethylphenol—and three dibasic phenolic compounds, including resorcinol, 5-methylresorcinol, and 2,5-dimethylresorcinol. For toxicity testing, all eight phenolics were separated into three groups: a mixture of all eight phenols, a mixture of the five monobasic phenols, and a mixture of the three dibasic phenols. The toxicity and biodegradability of these three groups of phenols were examined in three organisms, such as *Vibrio fisheri*, *Daphnia magna*, and *Thamnocephalus platyurus*. Phenol, p-cresol, resorcinol, and 5-methylresorcinol were the most rapidly detoxified after conventional activated sludge treatment. Dimethylphenols and the combinations required medium detoxifying times, while the 2,5-dimethylresorcinol and the resorcinol mixture were detoxified at the slowest rate. Among the eight phenolic chemicals examined, 2,5-dimethylresorcinol exhibited the highest toxicity and was classified as the most environmentally hazardous pollutant [37].

## 3. Physicochemical Methods of Phenols Treatment in Wastewater

Wastewater should be cleaned of toxic phenolic compounds to protect human health and the environment. A range of conventional and cutting-edge treatment methods are used to remove phenols from industrial effluents. These treatments are based on physical or chemical treatments including distillation, solvent extraction, evaporation, adsorption, oxidation, ion exchange, phytoremediation, and biodegradation [38,39,40,41]. 

Removal of phenols by distillation is based on the variation in steam distillation, which requires high energy levels; therefore, it is not economically feasible. This method can be either destructive or non-destructive, the latter of which enables the recovery of phenols. Steam or azeotropic distillation can remove phenolic pollutants from wastewater depending on the relative volatility of the substances [6]. In a study, an advanced steam plasma torch was used to directly inject an aqueous phenol solution. Phenols were quickly decomposed in the thermal plasma jet, generating hydroxyl radicals, which lead to oxidatively degrading organic pollutants in the aqueous solution. Pyrocatechol, hydroquinone, maleic acid, butanedioic acid, and muconic acid were the main liquid intermediates generated from phenols, whereas H_2_, CO, and CO_2_ were the main gaseous products. Steam thermal plasma technology is popular in the field of environmental cleanup due to its high thermodynamic efficiency, reactivity, and environmental friendliness [42].

Phenols can be removed from wastewater through adsorption or extraction techniques depending on the economy of utilizing and reusing the necessary secondary materials, i.e., adsorbent, or extractant. This technique is applied when the concentration of phenols ranges from a trace to a high percentage [43]. Phenol removal from wastewater through adsorption has been carried out with several types of adsorbents, including activated alumina [44], bentonite modified with a surfactant [45], activated carbon from biomass, organo-modified montmorillonite (MT) [46], titanium dioxide [47], and Filtrasorb-400 [48,49,50]. The most popular and effective adsorbent is activated carbon. The high specific surface area, tunable mesopore ratio and ease of procurement make activated carbon an outstanding candidate material with a high adsorption capacity for phenol removal from wastewater. Both physical and chemical activation methods, such as heating and nitric acid activation, can be used to improve the properties of activated carbon [51,52,53]. Recently, new methods have been developed, such as chemical modification of the activated carbon [54], impregnation with nanoparticles as magnetic activated carbon–cobalt nanoparticles, and silver nanoparticle-modified palm kernel shell activated carbon [55,56]. Different carbon sources, such as activated carbon prepared from biomass materials and avocado kernel seeds [57,58], using different activation methods [59], as well as substitution with affordable biosorbents such as chitin and chitosan, peat, and biomass [60] are promising alternatives for removing phenolic compounds. In a previous study, activated carbon from black wattle bark waste was produced under different carbonization and activation conditions for phenol adsorption. The highest adsorption capacity was 98.57 mg g^−1^ at 55 °C [61]. In the adsorption method, phenol is selectively deposited onto a solid adsorbent rather than being totally removed from wastewater. Therefore, it generates a lot of solid waste, which needs to be disposed safely. Using activated carbon for adsorption is, thus, expensive because of the recovery of activated carbon particles from the treated wastewater [62].

Another non-destructive method for treating phenolic compounds is liquid–liquid extraction, also called conventional solvent extraction. This method is acceptable throughout a wide range of phenol concentrations and provides economic feasibility in some cases [63,64]. In a study, imidazole and its similar compounds were used as extraction agents for liquid–liquid extraction to separate phenols from coal tar. It was reported that both imidazole and its homolog compounds could form a deep eutectic solvent with phenols, and, therefore, the removal efficiency was more than 90% [63]. In another study, Patel et al. applied liquid–liquid extraction for the treatment of pharmaceutical wastewater. They reported the maximum phenol removal efficiency as 68% using toluene as an organic solvent extractant [64]. The effectiveness of cumene as an extractant for phenol in wastewater was examined by Liu et al. for treating 100 mg L^−1^ aqueous phenol solution. It was reported that the stripping efficiency was above 99% when 0.1 mol L^−1^ NaOH was used for stripping phenol from loaded cumene [65]. The recovery and regenerating processes of extractant still involve considerable effort and are costly.

Ion exchange is another physicochemical method for phenolic wastewater treatment. A non-functional or mostly OH group ion exchange resin is used. It is documented that the resin type affects the phenol removal efficacy. Nevertheless, the ion exchange method of wastewater treatment is expensive because of the resin’s high cost. Furthermore, each resin removes specific pollutants. According to reports, non-functionalized ion exchange resins remove phenol more effectively in a basic medium than in acidic condition. Phenolic compounds are effectively removed by Amberlite XAD resins, which are hydrophobic polyaromatic resins with molecular weights ranging from 20,000 to 60,000 MW. Their non-polar to moderate polar property is suitable for the adsorption of both the non-polar aromatic structure and the -OH polar group of phenols. Previous studies showed that the sorption capacity of Amberlite XAD was higher for chlorophenol (2.27 mmol g^−1^) than phenol (1.50 mmol g^−1^). Resin regeneration was conducted through extraction (elution) with methanol [66,67]. After every process, the recovery and regeneration of the resin are relatively laborious and expensive.

In a destructive treatment, aqueous phenols are subjected to chemical oxidants. The most common chemicals used in oxidative wastewater treatments are ozone, chlorine, chlorine dioxide, chloramines, ferrate (FeO_4_^2−^ or Fe(VI)), and permanganate (MnO_4_^−^ or Mn(VII)). Because of their high reduction potential, permanganate and ferrate have received much attention [68,69,70]. Electrochemical oxidation is another destructive method of removing aqueous phenols that does not require additional chemicals but does involve energy and equipment costs. This method of phenol treatment is further divided into direct and indirect oxidation [71]. For direct electrochemical oxidation, contaminants are adsorbed onto the anode surface, resulting in direct or anodic oxidation. A variety of anode materials are used in direct oxidation; however, the most efficient one is boron-doped diamond (BDD) [72]. Besides BDD, other materials used in electrochemical oxidations are Pt, PbO_2_, SnO_2_, and IrO_2_, with the latter being the most explored due to its less bio-toxicity [73,74,75]. Indirect electrochemical oxidation uses intermediary redox chemicals to transfer electrons from the electrode to the pollutant, preventing the electrode from contamination and fouling. Indirect electrochemical oxidation with active chlorine is more effective compared to direct electrochemical oxidation as chloride enhances electron transport from the phenolic chemicals to the electrodes [76]. Based on the redox reagents, indirect electrochemical oxidation can be separated into two processes: anodic processes and cathodic processes. The cathodic process takes place at the cathode, where hydrogen peroxide (H_2_O_2_) is produced through the iron catalyst on the electrode surface, which is constantly renewed. Oxygen or air is continuously delivered to the area around the cathode to produce H_2_O_2_ [77]. This procedure is called electro-Fenton reaction (EF). The idea of EF has now been adopted by new emerging technologies, such as photo-EF and solar-EF, which combine the EF process with photocatalysis [78,79]. 

Although chemical oxidation processes have a number of advantages, the high cost of the chemicals and the emission of numerous toxic byproducts and harmful gases are the main problems. Several oxidizing agents, like hydrogen peroxide, have limited reactivity, resulting in the incomplete oxidation of many organic pollutants. Therefore, the focus of the present research has shifted towards technology that prioritizes phenol detoxification and degradation without the aforementioned downsides. Biological degradation utilizing both pure and mixed microbial strains has markedly become an appealing and practical alternative for the treatment of contaminated wastewater, including resistant substances like phenols, because the process is more selective, produces non-toxic byproducts and is economically feasible relative to the other aforementioned technologies [80,81].

## 4. Biodegradation of Phenols and Their Derivatives

Compared with the various treatment methods of phenolic chemicals in wastewater, biodegradation is the most effective. Biodegradation techniques have the ability to transform contaminated and toxic compounds in wastewater into safe and usable forms. It is a more affordable, sustainable, and environmentally friendly process. Several microorganisms such as bacteria, fungi, yeast, and algae have shown the ability to break down phenolic compounds under aerobic and anaerobic conditions. These microorganisms utilize phenols as a carbon source for their growth and metabolism. A range of microorganisms degrading various phenolic compounds are shown in Table 1. The *Pseudomonas* sp. is well known for its capacity to use a variety of aromatic compounds as a single carbon source. *Pseudomonas putida* exhibited a substantial degrading ability toward phenol and its derivatives like 2,4-dichlorophenol [82]. Mahgoub et al. isolated and identified three bacteria from sewage sludge, namely, *Pseudomonas aeruginosa*, *Klebsiella pneumoniae*, and *Klebsiella variicola*. They reported that these bacteria effectively degraded 1000 mg L^−1^ phenol in a mineral salt medium [83]. In addition to *Pseudomonas* sp. and *Klebsiella* sp., a number of bacterial species, including *Bacillus stearothermophilus* [84], *Bacillus laterosporus* [85], *Rhodococcus erythropolis* [86], and others, have been identified for their capacity to break down phenolic compounds. A variety of aromatic chemicals can be assimilated by these bacteria, though at low concentration. *Pseudomonas cepacia* and *Bacillus brevis*, which have been isolated from an industrial effluent which contains phenol, exhibit a high phenol degradation efficiency. The modified cultures of *P. cepacia* and *B. brevis* have been shown to break down 2.5 and 1.75 g L^−1^ of phenol in 144 h, respectively [87]. In another study, phenolic wastewater treated by *Acinetobacter calcoaceticus* lowered phenol concentration by 91.6% from 1.7 to 0.8 g L^−1^ after two days [88]. *Acinetobacter lwoffii* NL1 has a potential for effective phenol degradation in wastewater containing heavy metals since it can withstand up to 1.1 g L^−1^ of phenol and can break down phenol to a final concentration of 0.5 g L^−1^ in about 12 h [89]. A diverse consortium of immobilized microorganisms has been used in a nitrifying system to remove bisphenol A (BPA). In this study, with 1.5 h of hydraulic retention time (HRT), BPA was removed from an initial concentration of 10 mg L^−1^ by more than 92% [90]. *Pseudomonas putida* displayed a breakdown of 1 g L^−1^ phenol in 162 h (6.17 mg L^−1^ h^−1^) [91], while *P. cepacia*, isolated from industrial wastewaters, effectively degraded 2.5 g L^−1^ of phenol in only 144 h (17.36 mg L^−1^ h^−1^) [87]. At 0.5 g L^−1^ phenol, *Rhodococcus aetherivorans* even more degraded phenol at a rate of 35.7 mg L^−1^ h^−1^ [92]. The mutant M1 of *Rhodococcus ruber* SD3 displayed 98% phenol breakdown of a 2 g L^−1^ initial concentration in 72 h by immobilized cells system (27.2 mg L^−1^ h^−1^) [93]. *Klebsiella oxytoca* degraded 100 ppm phenol in 72 h [94]. In another study, a number of microorganisms were applied in the biodegradation of phenol to catechol. The degradation of β-naphthol was evaluated using *Volvox aureus*, *Lyngbya lagerlerimi,* and *Nostoc linckia*, while the oxidation of catechol was assessed using *Chlorella vulgaris* and *V. aureus*. The maximum naphthalene degradation was observed with *N. linckia* at 47.71% after 7 days, while anthracene was degraded at 92.28% by *E. viridis* after 7 days [95]. The separate and simultaneous biodegradations of phenol and *p*-cresol were evaluated using *Pseudomonas putida* ATCC 17484 in an aerobic batch reactor system. The concentration of phenol and *p*-cresol was in the same range as 50–600 mg L^−1^. Complete degradation of phenol and *p*-cresol was achieved within 48 h and 48–56 h, respectively, for all the initial concentrations of phenol and *p*-cresol [96]. Li et al. reported an almost 100% removal of 2,4-dichlorophenol (200 μg L^−1^) by *Chlorella pyrenoidosa* [97]. In another study, it has been revealed that bisphenol A can be adsorbed by algal cells, *Desmodesmus* sp. WR1. As a result, the efficacy of BPA removal may be higher due to algal bioactivity. BPA was incubated with algal cells, and BPA removal efficiencies were reported as 57%, 25%, 18%, and 26% for initial BPA concentrations of 1, 3, 5.5, and 13.5 mg L^−1^, respectively [98]. In case of bacterial degradation of phenols, both pure and mix cultures can be applied to phenol biodegradation [99,100]. However, high salt and phenol concentrations inhibit the growth of microorganisms by the mechanism of substrate inhibition. A high concentration of phenol also prevents microbe activity (or inactivation), which limits the efficacy of biodegradation. This problem greatly restricts the use of biodegradation methods in the treatment of phenol-rich and salty phenol effluents [101,102,103]. Therefore, it is particularly important to isolate suitable microorganisms that can effectively decompose phenol at a high concentration of salt to remove the maximum amount of phenol from contaminated effluents.

The main limiting factor of biodegradation is the capacity of microorganisms toward salt tolerance. Halophilic microorganisms, especially halophilic archaea, are apparently appropriate for such a treatment as they generate extracellular polymeric substances (EPSs) that maintain the structural stability of bacteria [104]. An EPS is a complex high-molecular-weight polymer mixture, protecting bacteria from hazardous compounds. An EPS is made up of protein, polysaccharides, humic acid, phospholipid, and nucleic acid, and it is produced through secretion by microbes. Bacterial EPSs can absorb organic compounds through hydrogen bonds along with hydrophobic and electrostatic interactions. There have been studies on how EPSs affect the development of biofilms for the improved degradation of polycyclic aromatic hydrocarbon. Phenanthrene and pyrene biodegradation through EPSs in *Micrococcus* sp. PHE9 and *Mycobacterium* sp. NJS-P has been improved significantly [105]. It was demonstrated that EPSs act as electron shuttles because protein and humic compounds are highly enriched with conductive substances. Therefore, the overall electron transport system in EPSs is much greater than the normal electron transport chain. According to the previous studies, phenol degradation in bacteria like *Syntrophorhabdus* sp., *Brooklawnia* sp., *Treponema* sp., *Syntrophus* sp., and electroactive methanogens such as *Methanosaeta* sp. is markedly enhanced by the addition of conductive substances. Moreover, the addition of mediators such as aromatic lignin molecules assists the electron transporting system and reduces the notable chemical oxygen demand (COD) of wastewater containing phenolic compounds under anaerobic conditions [106,107,108]. The functional genera involved in methanogenic phenol degradation by means of extracellular electron transfer have been hypothesized for the *Syntrophus* sp. and the *Methanosaeta* sp. [104]. Furthermore, removing bacterial EPSs drastically reduces the organic absorption ability of microbes. These findings suggest that EPSs are crucial for the uptake and removal of organic substances during biodegradation. However, there is still less research on the function of EPSs and their release during phenol biodegradation [109,110,111].

## 5. Mechanism of Phenol Biodegradation

### 5.1. Aerobic Biodegradation

The mechanism of phenol degradation was first identified in the 1950s, and it is now well established. Many aerobic phenol-degrading bacteria have been discovered during the last few decades. A vibrio-like organism called ‘Vibrio 01′ has been isolated from wastewater discharged during coal gasification. It was firstly reported for its synthesis of beta-ketoadipate, which is an intermediate of phenol biodegradation [112,113]. Then, a number of microorganisms, including *Pseudomonas species*, *Acinetobacter calcoaceticus*, a thermophilic *Bacillus* species, an actinomycete *Streptomyces setonii*, and two eukaryotic microorganisms, the yeasts *Trichosporon cutaneum* and *Candida tropicalis,* have been isolated from phenol degradation system [114].

**Figure 1:**
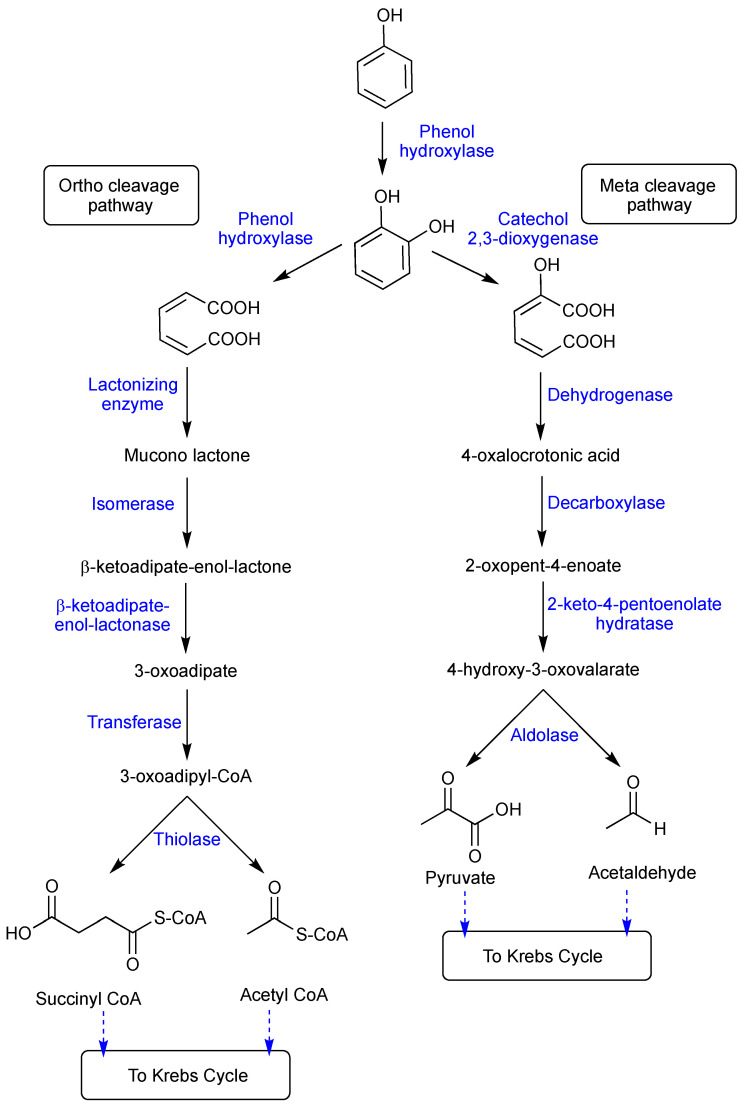
Ortho and meta pathways of aerobic biodegradation of phenol and its derivatives in microorganisms [116]. Copyright 2016, Elsevier. Note: Texts with black color are degrading substances and those with blue colors are enzymes.

The oxygenation of phenol is the first step in the aerobic breakdown of phenol by microorganisms. This step is catalyzed by phenol hydroxylase, which is a mono-oxygenase enzyme involved in the aerobic phenol degradation pathway (Figure 1). In this step, a catechol is generated by the phenol hydroxylase-catalyzed mono-hydroxylation of the phenol’s aromatic ring at the ortho position to the hydroxyl group. This is the main output of the breakdown of phenol by various microorganisms. Catechol then goes through ring cleavage, which depends on the microbial strain. It can occur in either ortho or meta positions, triggering either the ortho pathway which generates succinyl Co-A and acetyl Co-A or the meta pathway which produces pyruvate and acetaldehyde. *Pseudomonas putida*, Pseudomonas cepacia, Pseudomonas picketti, and *Alcaligene deutrophus* have been reported for their phenol biodegradability via the meta cleavage pathway, while *Trichosporon cutaneum*, Rhodotorula rubura, and *Acinetobacter calcoacetium* have been discovered for their biodegradability via the ortho cleavage pathway [115]. The mechanism of the aerobic degradation of phenol and its derivatives in microorganisms is shown in Figure 1.

### 5.2. Anaerobic Biodegradation

**Figure 2:**
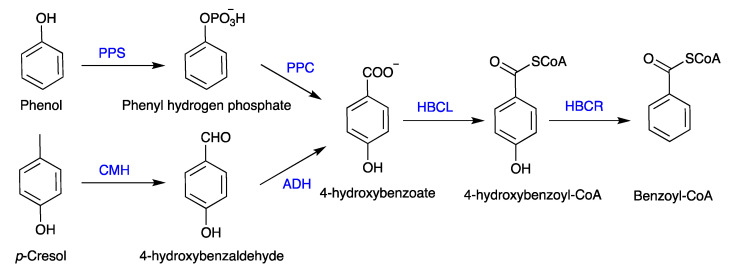
Anaerobic biodegradation pathway of phenol and *p*-cresol in bacteria [117]. Copyright 2015, Elsevier. Phenyl phosphate synthase (PPS); *p*-cresol methyl hydroxylase (CMH); phenyl phosphate carboxylase (PPC); aldehyde dehydrogenase (ADH); 4-hydroxybenzoate-CoA ligase (HBCL); and 4-hydroxybenzoyl-CoA reductase (HBCR). Note: Texts with black color are degrading substances and those with blue colors are enzymes.

Although anaerobic biodegradation is less effective than the aerobic process, phenol can nevertheless be biologically degraded in the absence of oxygen. Carboxylation at the para position of phenol to 4-hydroxybenzoate is the first step in the anaerobic phenol breakdown pathway (Figure 2) [117]. The anaerobic pathway exhibits two processes of phenol carboxylation. The enzyme phenyl phosphate synthase (kinase) initially phosphorylates phenol by adding a phosphate group from an unidentified phosphoryl donor, which catalyzes the formation of phenyl phosphate as the first intermediate. Subsequently, the enzyme phenyl phosphate carboxylase, which requires Mn^2+^, carboxylates phenyl phosphate to produce 4-hydroxybenzoate. The synthesis of phosphorylation and carboxylation enzymes is strictly regulated.

The anaerobic biodegradation of phenol involves several intermediates, including benzoate, catechol, cis-muconate, α-ketoadipate, succinate, and acetate [118]. It has been intensively studied on how pure and mixed microbial cultures degrade phenol. The *Pseudomonas* sp. has been mostly used in various studies on phenol breakdown. Phenol can be degraded to a free form or bound to soil or sediments, even though the rate of biodegradation is slow down in the presence of a sorbent [119]. The phenol carboxylation enzyme (phenyl phosphate carboxylase) in *Thauera aromatica* seems to proceed through a phosphorylated-free intermediate and is not related to any of the studied carboxylases. Phenyl phosphate carboxylase has a high sensitivity to oxygen and is receptive to molecules that trap free radicals but not dependent on biotin or thiamine diphosphate. Unlike the other known carboxylases, it uses metal as a co-catalyst and carbon dioxide as a substrate [120,121]. *Rhodopseudomonas palustris*, *Magnetospirillum* sp., *Thauera aromatica*, *Azoarcus* sp., *Geobacter metallireducens*, and *Syntrophus aciditrophicus* were the first group of anaerobic phenol-degrading bacteria explored for chemical and genomic characterization [122,123]. In a laboratory and pilot-scale investigation, several bacterial species were found in anoxic granular denitrifying reactors for the treatment of synthetic wastewater containing phenol as the only carbon source because of their capacity to utilize aromatic chemicals as electron donors. These bacteria included *Desulfotomaculum* sp., *Clostridium* sp., *Syntrophus* sp., *Ignavibacterium* sp., *Denitratisoma* sp., and *Thaurea* sp. [124,125]. Along with *Advenella* sp., *Corynebacterium* sp., *Sphingobium* sp., and *Ottowia* sp. were listed as the most prevalent genera of phenol degraders for refining wastewater [126]. The anaerobic biodegradation pathway of phenol and *p*-cresol in bacteria is shown in Figure 2.

## 6. Membrane Bioreactors (MBRs)

The biological approaches for wastewater treatment have been used since long time ago. Activated sludge is the most common biological treatment method applied in wastewater treatments [127]. When a direct reuse of effluent or a strict discharge requirement are intended, filtration techniques like reverse osmosis, ultrafiltration, microfiltration, and nanofiltration are widely used. The MBR system, therefore, is currently gaining popularity because of advancement in highly efficient membrane technology and the availability of selective membrane modules [128,129].

**Figure 3:**
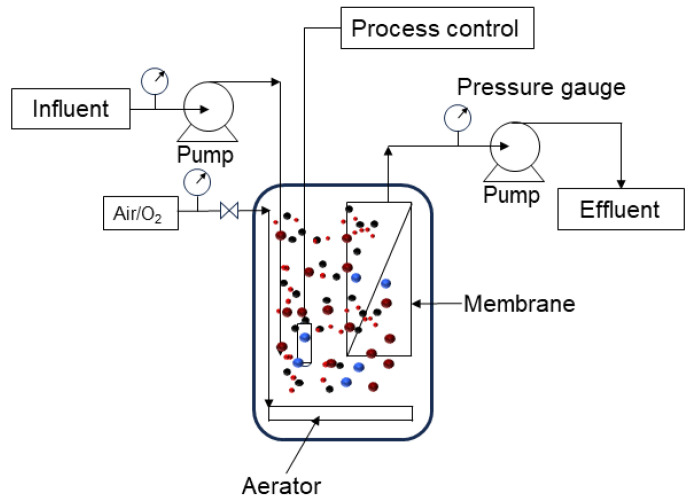
An integrated (internal) MBR system [134]. Copyright 2018, Elsevier.

Generally, an MBR is made up of two main components: (1) the biological unit, which is involved in the biodegradation of wastewater; and (2) the membrane module, which physically separates treated water from mixed wastewater [130,131]. Depending on the configuration, MBRs can be divided into two categories: integrated MBR systems and recirculated MBR systems. The bioreactors equipped with internal membranes are included in the first category, which is commonly called an integrated MBR system (Figure 3). The driving force across the membrane is produced by pressurizing the bioreactor or generating negative pressure on the permeate side. Frequent permeate back-pulsing and sporadic chemical backwashing are used to clean the membrane. To assist with the scouring of the filter surface, a diffuser is positioned directly beneath the membrane module. At the same time, the diffuser is simultaneously utilized for mixing and aeration purposes. Anaerobic or anoxic compartments can be added to allow the simultaneous biological removal of substrates [132,133].

**Figure 4:**
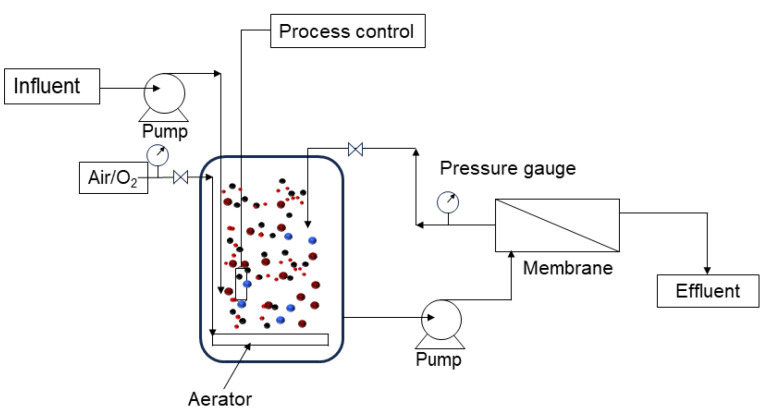
A recirculated (external) MBR system [134]. Copyright 2018, Elsevier.

The second configuration is known as a recirculated (external) MBR system, in which the membrane module is externally assembled to the bioreactor (Figure 4). Membranes for both the inner and outer skin layers can be utilized in this application. A high crossflow velocity of the feed along the membrane surface results in a pressurized environment, which acts as a driving force for the separation. The commercial use of MBRs has been increasing currently due to the development of less expensive, and more durable polymeric membranes such as polyvinylidene fluoride (PVDF), polyether sulfone (PES), polyethylene (PE), and polysulfone (PSF) that provide lower pressure requirements, and larger permeate fluxes [133,135].

A MBR system has numerous benefits over conventional activated sludge (CAS) and other wastewater treatment methods. Primarily, the bioreactor’s ability to retain all the suspended matter and most of the soluble substances results in an outstanding effluent quality that may pass strict discharge standards and pave the way for direct water reuse. Secondly, MBRs have the potential to retain microorganisms e.g., bacteria and viruses, especially pathogenic microbes in the bioreactor, leading to a sterile effluent and reducing the requirement of thorough disinfection and associated health risks. The best possible management of microbial population control and operational flexibility is accomplished by the fact that the clarification stage does not cause any losses of suspended solids. The total separation and the control of solid retention time (SRT) and hydraulic retention time (HRT) are, therefore, achievable. With a lack of clarifier, which also acts as a natural selector for settling microorganisms, the anaerobic MBR system enables delicate and slow-reproducing species to grow. For example, denitrifying bacteria, which are able to digest complex compounds, emerge and persist in such a system even during brief SRTs [18,136,137]. Common denitrifying bacteria such as *Acinetobacter* sp., Azoarcus sp., Thauera sp., Acidovorax sp., and *Stenotrophomonas* sp. are employed for phenolic treatment in wastewater [138,139,140]. Settling is a major challenging aspect of wastewater treatment that is typically eliminated with MBRs [141,142]. High microbial concentrations are possibly employed in an MBR system due to its capability of operating at very high solid retention durations without encountering the problem of settling. The quality of the rejected water is unaffected by changes in the sludge settling velocity because of the membrane module’s improved compactness compared to a conventional secondary clarifier [143]. Suspended solids can be totally removed by using the membrane to separate liquid and solid components rather than relying on the traditional method of settling [144,145] in which the sludge concentration and hydraulic loading rates are higher that cause increased expenditures. Input is pre-screened and processed before entering in the MBR, where biodegradation takes place [146,147,148]. The mixed fluid in the bioreactor is removed and pumped along modules with submerged or semi-crossflow filtering membranes. Concentrated biosolids from the reject stream are fed back into the bioreactor [149,150]. Excess biosolids from the reactor or the return line are discarded. The separation of the HRT from the solids’ retention time offers excellent control of the biological activities. Due to the high biomass concentration in MBR, the system is substantially smaller than a conventional wastewater treatment system [151,152,153]. 

The MBR is a hybrid approach that combines membranes with the traditional biological process. Initially, the MBR system was applied to domestic wastewater treatment but has been now effectively used in the treatment of industrial effluents containing phenolics [6,154]. Due to its low carbon emission, the MBR system has attracted much attention for its wastewater treatment applications. The advantage associated with the MBR over other treatment techniques is disinfection. The biological process that is part of the MBR system contains microbes and activated sludge [142] that effectively degrade hydrocarbon, including phenol, through their microbial metabolism. The parameters for biodegradation, such as pH, oxygen, temperature, nutrient, and the uniform distribution of biomass and pollutants in the reactor, are more precisely controlled and managed by the bioreactor component. The MBR system enables longer sludge retention times and higher mixed liquor suspended solid concentrations than traditional activated sludge, hence allowing the biomass to develop and adapt toward wastewater more effectively [155]. 

Wastewater first undergoes pretreatment or screening to remove grit, hair, and other fibrous and abrasive contaminants that might clog the membrane and cause a significant and quick decrease in flux. Therefore, a multi-step physical process including grit removal and fine-screening is necessary before entering the MBR system. Air is then introduced to scour the membrane and propel the biological treatment to break down hydrocarbon. To prevent membrane fouling and force the water to pass through the membrane, which serves as a solid–liquid separation mechanism and keeps the microbial inside the bioreactor, a significant amount of pumping power is needed to maintain high velocity, pressure, and flux. Disinfection is applied to kill microorganisms that are retained in the bioreactor [156,157].

## 7. Modified Membrane Bioreactors (MMBRs)

Compared to traditional treatment methods, modified membrane bioreactors (MMBRs) have several benefits in wastewater treatment. The comparison of phenol removal by different configurations in membrane bioreactors is shown in Table 2. MMBRs clean wastewater very effectively because of their combination of biological processes and membrane filtration. MMBRs have a smaller environmental impact than traditional wastewater treatment methods as secondary clarifiers are not necessary with the membrane filtering method, enabling the design of a more streamlined and compact treatment facility. This is especially helpful when retrofitting existing treatment facilities or in places with small area available. This can lessen the environmental impact of sludge management and lower the expenditures related to sludge handling and disposal [158]. The membrane filtration in MMBRs offers excellent solid removal, results in effluent with a low turbidity and fewer suspended solids. Furthermore, the membrane barrier effectively retains bacteria and pathogens, leading to a high level of microbial removal. As a result, effluent from MMBRs is often of superior quality, meeting strict water reuse criteria and reducing the risk of waterborne diseases. Advanced monitoring and control systems can help modified MMBRs with the real-time monitoring of numerous parameters, such as membrane fouling, oxygen levels, pH or nutritional concentrations and gaseous products. This enables operators to improve system performance real-time, identify problems quickly, and choose wisely between necessary maintenance and operational changes. Thus, MMBRs are a desirable alternative for both municipal and industrial wastewater treatment applications because they generate high-quality effluents that can be used for a variety of water reuse tasks, including irrigation, commercial operation, and groundwater replenishment [159,160].

### 7.1. Capillary Membrane Bioreactor (CMBR)

**Figure 5:**
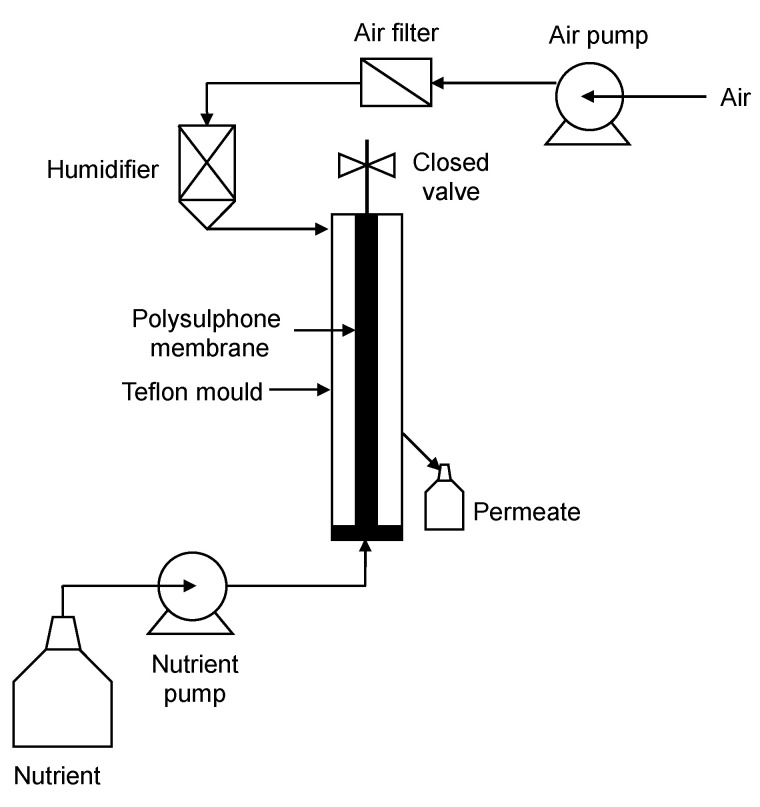
A single-fiber immobilized cell, capillary membrane bioreactor system [177]. Copyright 2006, Elsevier.

A capillary membrane bioreactor has been studied and tested for the removal of phenolic compounds from synthetic and industrial effluents (Figure 5). Two polymeric membranes with various morphologies have also been used to immobilize polyphenol oxidase on a single capillary membrane in a small-scale bioreactor. The uniqueness of this membrane is its lack of the outer skin layer that generally serves as a support. Therefore, it permits a higher flow rate and has demonstrated remarkable performance in the removal of substances from this kind of reactor. It has been reported that 45 units of polyphenol oxidase were used with this high-flux membrane to remove 949 µmol of phenols from a solution containing 4 mmol L^−1^ of total phenolics in 8 h. This result was much greater than the 120 µmol of phenolics that was removed using a non-immobilized enzyme, as the enzyme had become inactive due to product inhibition after 7 h [174]. Oxidative enzymes, produced by the fungus *Neurospora crassa*, have been utilized in the biodegradation of phenolic compounds in both shaken and static batch cultures equipped with a capillary membrane. In the above-mentioned research, the *N. crassa* enzyme system was used to execute bioremediation on two phenolic substrates, phenol and *p*-cresol, which are components of effluents discharged from industries. Over a six-day period, the fungal cultures in the flasks removed 18 mg *p*-cresol and 23 mg phenol, respectively, from the initial 5 mmol L^−1^ solutions of g^−1^ wet biomass. While the immobilized cultures’ system removed 10 mg *p*-cresol or 8 mg phenol g^−1^ wet biomass during the same period. It was reported that the immobilized biomass in a continuous reactor could maintain this removal efficacy for a duration of four months. In contrast, the batch liquid culture systems-maintained activity for an estimated 8–15 days, after which the cultures were no longer viable. This was the first instance of continuously applying immobilized *N. crassa* biofilms for phenol bioremediation [175]. In a study, Pseudomonas putida was used in an immobilized cell membrane bioreactor to degrade large concentrations of phenol. It was documented that *P. putida* in suspension cultures utilized phenol at concentrations below 1000 mg L^−1^, but it experienced substrate inhibition at higher concentrations. On the other hand, *P. putida* immobilized in 25 w% polysulfone fibers decomposed phenol at a higher concentration. The immobilized cells completely broke down the phenol within 9 h at a phenol concentration of 1200 mg L^−1^; however, no cell growth or phenol degradation appeared in the free-suspension system at 1000 mg L^−1^ phenol. In a number of studies, it has been noticed that cells dispersed from the membranes once phenol concentrations reached sub-inhibitory levels. In these instances, the duration required for full degradation was reduced when cell diffusion was employed, as quick phenol degradation was primarily attributed to the suspended cells [176]. An example of fifteen single-fiber capillary membrane bioreactor (SFCMBR) setup for kinetic study of *Phanerochaete chrysosporium* biofilms was illustrated in Figure 5. This bioreactor units were vertically oriented according to the configuration and the capillary polysulphone membranes utilized were internally skinned and externally unskinned, manufactured specifically for this purpose [177].

In another study, chitosan gel was chosen as a matrix to coat on skinned polysulphone capillary membranes for the immobilization of polyphenol oxidase. The effect of this chitosan coating on the removal of phenolics from industrial wastewater by immobilized polyphenol oxidase was studied using bench-scale single-capillary membrane bioreactors. This study showed that capillary membranes coated with a gel-like chitosan exhibited higher protein carrying capacities than uncoated membranes. Due to the action of chitosan, which provides an in-situ product removal function along with a greater enzyme loading capacity. The enzyme loading capacity for the chitosan-coated membrane was 143 U with a phenol removal of 1224.4 mg, which was remarkably high compared to the non-coated capillary membranes, for which the values were 55 U and 20.3 mg, respectively. Thus, the use of immobilized polyphenol oxidase and chitosan-coated capillary membranes serves two purposes: first, they provide a highly effective method for removing phenolic contaminants from water, and second, they provide a method for successfully removing color from the resulting permeate. Furthermore, the presence of chitosan significantly reduces the product inhibition that is a characteristic of polyphenol oxidase [178].

### 7.2. Extractive Membrane Bioreactor (EMBR)

**Figure 6:**
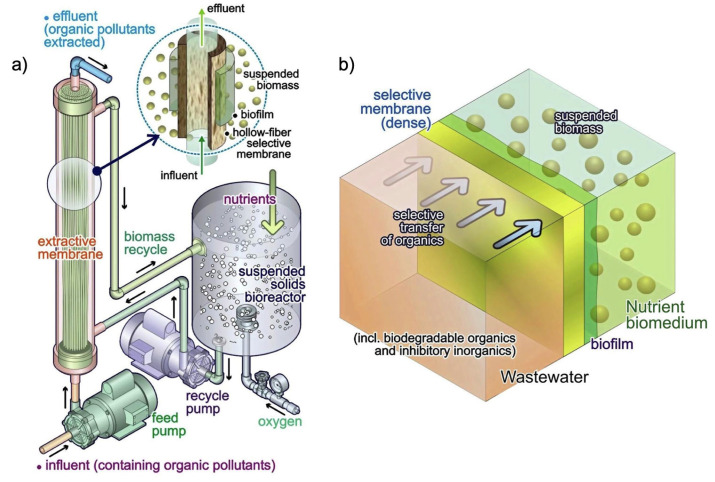
(**a**) EMBR and (**b**) principle of EMBR adopted from [180]. Copyright 2020, Elsevier.

The extractive membrane bioreactor (EMBR) is a promising technology used to treat wastewater, like in the processes of denitrification of drinking water, organic saline removal from wastewater, and organic pollutant removal [179,180]. As shown in Figure 6a, an EMBR is made up of three parts: an effective microorganism compartment, a selectively permeable membrane, and a targeted effluent compartment. This system allows the separation of biomass from harmful wastewater, provides concentration gradient control, and yields a slow release of pollutants into the bioreactor through an absorptive or diffusive membrane where microorganisms break down organic molecules. The membrane only permits the passage of target chemicals depending on their thermodynamic affinities [181]. The EMBR separates organic chemicals from inorganic compounds while simultaneously facilitating their biological degradation. The selective membrane helps the EMBR to remove particular extracted molecules from the waste stream. The hollow-fiber selective membrane allows the selective transport of biodegradable organic pollutants through the membrane and biofilm from the lumen into the shell side. The bio-medium is circulated on the shell side, while the wastewater is pumped into the membrane module through the lumen side of the operating mechanism. Subsequently, only the target components are introduced into the bio-medium [180]. The bulk solution of extracted organic molecules and biomass is progressively released to the other side of the membrane, and then it is conveyed to bioreactor in which biodegradation occurs [181]. 

In the EMBR, diffusion is the method by which mass is transferred (Figure 6b). In this setup, wastewater flows to the membrane module via the lumen side, while the bio-medium is recirculated via the shell side. When a nonporous membrane (such as polydimethysiloxane or PDMS) is utilized, the diffusion component is characterized as a solution-diffusion mechanism. For these kinds of membranes, components diffuse across the membrane from the feed side into the bio-medium after dissolving into the membrane from the feed side [180]. To supply vital nutrients and oxygen to the biofilm and eliminate biodegradation products like phenol, a small stream runs through the bio-medium. The biodegradation of the organic components contained in a hydraulic fracturing effluent has been investigated using an EMBR employing Hytrel^TM^3548 tubing. Methylethylketone, benzene, phenol, and acetic acid were present in significant amounts (1000 mg L^−1^) in the synthetic hydraulic fracturing effluent, along with 30–120 g L^−1^ of Cl^−^ at a low pH. This wastewater was pumped through the polymeric tubing, which then allowed an enhanced bacterial consortium of *Pseudomonas* sp., Comamonas sp., Achromobacter sp., Lysinibacillus sp., and *Oxalobacter* sp. to preferentially transport the organic chemicals across the membrane for biological breakdown. The report showed a 99% removal of benzene and phenol, a 96% removal of methylethylketone, and a 53% removal of acetic acid by a continuous EMBR operation [162]. 

An EMBR can be operated at ambient temperature and pressure, displaying a wide range of applications and being appropriate for wastewater containing harsh or inhibiting substances such as salinity, acid, or alkali substances. During biodegradation, isolated organic components are completely broken down, necessitating no further generation of pollutants. The EMBR is primarily utilized for water rejection and organic compound permeation, as opposed to nanofiltration (NF), reverse osmosis (RO), and forward osmosis (FO), which are often used for organic compound rejection and water permeation.

Based on the three hypotheses which form the foundation of the models, the mass transfer coefficient may be defined using each of the membrane’s distinct barriers. First, the mass transfer is in a steady-state condition. Second, the membrane’s pores contain the organic aqueous liquid interface. Third, the coefficients of solute partition across the concentration range are assessed using the two liquids’ immiscibility. Using EMBRs, numerous studies have been carried out to remove phenolic compounds from wastewater. In a study, an EMBR membrane made of polydimethylsiloxane, polymethyl methacrylate, and multi-walled carbon nanotubes was employed. In comparison to those without activated sludge, the phenol transmembrane mass transfer rates rose by 21.6–31.7% when biofilm was formed and activated sludge was present. With 1000–4000 mg L^−1^ phenol and 10 g L^−1^ sodium chloride in the effluent, 100% of the phenol was removed, while 99.96% of the salt was rejected [161]. Another study showed a novel EMBR for simultaneous phenol permeation, salt rejection, and biodegradation. The electro-spun polydimethylsiloxane/polymethyl methacrylate (PDMS/PMMA) membrane showed contact angles of 160.9 ± 2.2° for water and 0.0° for phenol, indicating that this superhydrophobic/super-organophilic membrane was suitable for separating phenol from water-soluble salt. Under an HRT of 24 h, phenols with concentrations ranging from 14.1 ± 2.7 to 290.7 ± 10.4 mg L^−1^ were continuously permeated and were totally biodegraded in an external EMBR, which corresponded to an improvement in detoxification performance from 6.3% to 70.5% [156]. A combination of phenolic chemicals, including benzene (513 ppm), toluene (269 ppm), ethylbenzene (78 ppm), and xylene (177 ppm), were metabolized in an EMBR by microbes *Pseudomonas putida* TX1 and BTE1. The EMBR system efficiently eliminated the phenolics at rates of up to 30 µg h^−1^ cm^−2^ of the membrane area, with an efficiency of removal ranging from 75% to 99% depending on the concentration of the phenolic compound and their vapor flow rate [163].

### 7.3. Hollow-Fiber Membrane Bioreactor (HFMBR)

**Figure 7:**
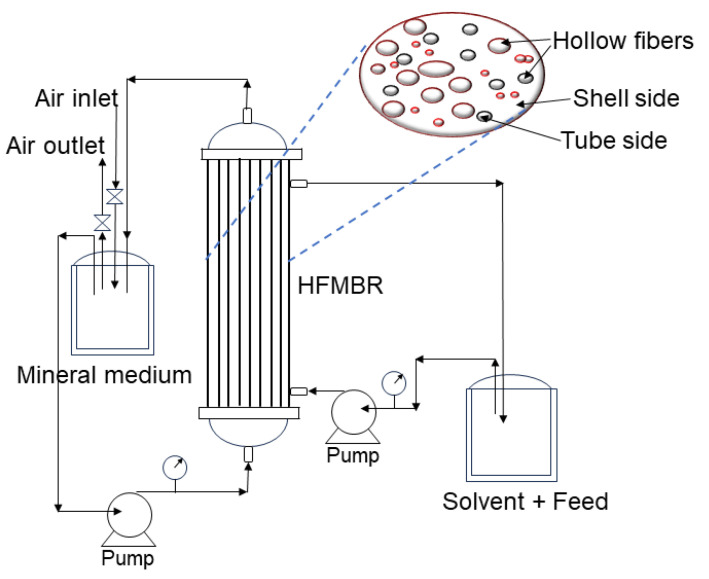
Flow diagram of an HFMBR system [184]. Copyright 2021, Elsevier.

The hollow-fiber membrane bioreactor (HFMBR) is an efficient membrane configuration for wastewater treatment because it has the maximum surface area per unit volume, as illustrated in Figure 7. The HFMBR is mechanically self-supporting and can encourage hollow-fiber movement through backwashing and air scouring, which aid in reducing membrane fouling [182]. HFMBRs are made up of hollow-fiber membranes that are parallel-assembled in an exterior shell with shape like a cylinder. The membranes are produced when the fibers are placed in the shell [183]. In a study, an HFMBR was developed for the biodegradation of phenol. Through mixing, solvent was consistently distributed throughout the feed phenol solution. Two peristaltic pumps were used to deliver the aqueous organic dispersion to the shell side, and the cell culture from the medium vessel to the tube side in the HFMBR as illustrated in Figure 7. HFMBR is equipped with carbon nanotube hollow-fiber membranes. A 0.45 µm filter was used to sterilize the cleaned air before it was introduced to a humidification tank. The cells were then sparged with the saturated air at two gas volumes per reactor volume per minute [184]. A flow diagram of the HFMBR is shown in Figure 7.

The HRT and the temperature are two main parameters that need to be closely monitored while operating a HFMBR. A short HRT may result in a high organic load rate, which is related to both the characteristics of the biomass used in the activated sludge process and the MBR process’s ability to treat wastewater effectively [185]. On the other hand, an extended HRT improves effluent quality by allowing bacteria for a longer time to degrade contaminants in wastewater [186]. Moreover, raising the temperature may result in a drop in feed viscosity and an increase in solute diffusion, which would raise the flux [186]. Aeration is another essential factor because it offers dissolved oxygen to biomass, which improves the efficiency of biodegradation [187]. Low aeration causes high salt concentrations in wastewater, which increase stressed conditions to microbes in a way which may have harmful or inhibitory effects to microorganisms. Environmental stress also makes bacteria undergo plasmolysis, lose cell function, or even develop too much EPS and soluble microbial products (SMP), which may block membrane pores and cause fouling [188]. Moreover, the properties of hollow-fiber membranes and their filtration efficiency are influenced by the membrane’s condition. The low hollow-fiber packing density, long and loose fibers, and small hollow-fiber diameter that allow the lateral movement of fibers enhance the hollow membrane’s filtration performance [182].

Trioctylphosphine oxide impregnated in polypropylene hollow-fiber membranes was used by Praveen et al. in an HFMBR system for the two-phase biodegradation of phenol employing *Pseudomonas putida* ATCC 11172. At a specific growth rate of 0.73 h^−1^, 1000 mg L^−1^ phenol was completely degraded in 12 h with the average biodegradation rate of 86 mg L^−1^ h^−1^. During the biodegradation of 3000 mg L^−1^ phenol, the biodegradation capacity and the degradation rate of the extractant impregnated hollow-fiber membrane bioreactor (EIHFMB) were enhanced by extending the effective length of the fibers by 50%. Increasing the aqueous phase flow rate also improved the adsorption and desorption rates [172]. Recently, the degradation and separation of purified terephthalic acid (PTA) wastewater in a lab-scale hollow-fiber anaerobic membrane bioreactor (HF-AnMBR) was examined by Kudisi et al. Synthetic PTA wastewater was run for 200 days with progressively lower HRTs to study long-term performance, membrane fouling mechanism, and the evolution of microbial communities. According to the published results, the methane generation rate was 0.33 ± 0.02 L L^−1^ reactor d^−1^, with a steady COD elimination rate of 65.8 ± 4.1% at an organic loading rate of 3.1 ± 0.3 g COD L^−1^ reactor d^−1^ and an HRT of 24 h. The reduced COD removal could have been related to the shortened HRTs. As a result, the petrochemical wastewater had a high level of toxicity, which inhibited the accumulation of harmful materials in the HF-AnMBR system and led to microorganism survival at risk. Consequently, methane formation was inhibited, and COD removal was reduced. The predominant methanogens that produced methane were *Methanosaeta* sp. and *Methanolinea* sp. [186]. In a different study, *Pseudomonas putida* was used for the biodegradation of phenol in an HFMBR containing granular activated carbon (GAC). In batch biotransformation experiments, the hybrid bioreactor completely removed 1000 mg L^−1^ phenol (at this concentration free cells cannot grow) in a hollow-fiber membrane bioreactor containing GAC in 18 h, compared with the 23 h taken in a GAC-free bioreactor. When phenol loading was <24 mg h^−1^, more than 90% of the phenol was converted during a continuous operation in the GAC bioreactor [173]. In another study, lithography was used to make polyvinylidene fluoride (PVDF) hollow-fiber modules with patterned membrane surfaces. The anti-biofouling characteristics of two distinct patterned hollow-fiber (PHF) PVDF membranes, a pyramid and a prism, were examined in an MBR that was used to treat wastewater. Due to the increased effective membrane surface, the PHF demonstrated a higher water flux as well as improved anti-fouling properties compared to the non-patterned hollow-fiber membrane [189].

### 7.4. Moving Bed Biofilm Reactor (MBBR)

**Figure 8:**
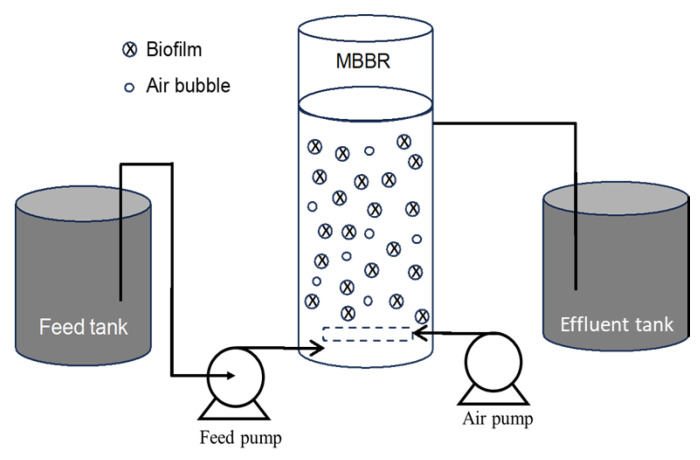
Flow diagram of a MBBR [165]. Copyright 2014, Springer Nature.

A moving bed biofilm reactor (MBBR) is a fully mixed continuously running biofilm reactor that was developed to provide the benefits of the biofilm process, including steady toxin removal. A larger biomass concentration can be obtained in MBBRs by using large moving media or media with a high effective biofilm surface area, which enhances toxin resistance and consequently boosts MBBR performance [190,191]. The other benefits of the MBBR technology include an enhanced volumetric treatment capacity, a low head loss, less clogging of the carrier medium, an increased resistance to environmental changes, reduced space requirements, and shorter HRTs [192,193]. The fundamental concept of the MBBR technology is that biomass is developed on uniquely designed carrier parts that can freely move throughout the reactor due to mechanical mixing, liquid recirculation, or aeration [194]. The flow diagram of an MBBR system is shown in Figure 8.

Several factors, including pH, nutrients, and HRT, affect the efficiency of MBBRs. Biofilm development is controlled by the availability of nutrients, and, hence, biofilm flourishes when the system contains a high number of nutrients. On the other hand, pH fluctuation influences the growth of biofilm since bacteria can modify the protein activity and protein synthesis related to various cellular processes during pH fluctuation [192]. A long HRT facilitates the acclimatization of microorganisms in wastewater, thus improving the removal efficiency of the pollutants. Conversely, a short HRT can lead to incomplete degradation. Increasing the wastewater loading rate over the biomass’s capacity for biodegradation may prevent complete mineralization. Therefore, the concentration of metabolites in the effluent could be increased [164]. Another factor influencing system performance is the biofilm carrier. In MBBR systems, biocarriers can be modified according to the categories of the process, namely, aerobic, anoxic, and anaerobic. A biocarrier with wider openings minimizes specific surface area loss, which makes it suitable for the rapid-growing heterotrophic biofilm in aerobic systems, whereas for slow-growing autotrophic microbial biofilm, a more appropriate biocarrier may contain small openings and a large effective surface [192]. Biocarriers, including polypropylene-polyurethane (PP/PU) foam [164] and polyethylene (PET) [165], have been successfully used to degrade phenol. 

In order to remove phenol and ammonia collectively, a MBBR containing biocarriers made of PP/PU foam was investigated by Swain et al. [164]. Retention time, pH, and air flow rate were the three variables studied. Under ideal circumstances, the maximal removal of phenol and ammonia was measured to be 92.6 and 91.8%, respectively. In another study, an immobilized *Bacillus cereus* GS2 biocarrier was used in a lab-scale MBBR. The response surface method was applied to optimize the process variables including the mixing intensity, the phenol content, and the HRT. At 100 rpm of mixing, 200 mg L^−1^ of phenol concentration, and 24 h of HRT, the best phenol removal efficiency (87.64%) was reported. The increased mixing intensity substantially improved the substrate diffusion between the liquid phase and the surface of the biofilm. Catechol and 2-hydroxymuconic semialdehyde were found to be produced during the biodegradation of phenol, providing additional proof that the *Bacillus* sp. followed the meta cleavage pathway (Figure 1) [166]. The effectiveness of an aerobic MBBR was assessed for the removal of sole phenol from saline wastewater. A mixed culture of active biomass that had been gradually acclimated to salt and phenol was introduced into the 10 L MBBR for phenol and COD removal. The studied parameters included inlet phenol concentration, HRT, inlet salt content, phenol shock loading, hydraulic shock loading, and salt shock loading. The findings showed that the HRT and the concentration of phenol and salt in the bioreactor feed have an impact on the removal efficiency of phenol and COD. At inlet phenol concentration up to 800 mg L^−1^, an HRT of 18 h, and inlet salt value up to 40 g L^−1^, the MBBR could remove up to 99% of phenol and COD from the feed saline wastewater. Additionally, quantifying biological factors revealed that the biofilm is a crucial factor for the removal of phenols [165].

Industrial wastewater having a low phenol concentration (8–16 mg L^−1^) and a high salinity (~150–160 mS cm^−1^) was treated in a membrane biological reactor with submerged flat membranes in both lab-scale and pilot-scale conditions. During the operation of both reactors, the phenol loading rate was gradually increased, and less than 1 mg L^−1^ phenol was detected even at very low HRTs (0.5–0.7 days). Membrane fouling was reduced by increasing the crossflow aeration rate within the MBR and using alternating permeation. A microbial community study of both reactors revealed that representatives of the genera *Halomonas* and *Marinobacter* were prominent. The phenol removal efficiency was >98.5% from the industrial hypersaline wastewater (8–15 mg L^−1^) with a short HRT (0.5 days) and an excellent operational flexibility [167]. In another study, phenol-rich pharmaceutical wastewater was treated in membrane bioreactors by applying quorum quenching (QQ) technology to minimize membrane fouling. *Rhodococcus* sp. BH4 and an isolated quorum quenching consortium (QQcs) from activated sludge were used for reduction of membrane fouling in MBR. It was shown that neither BH4 nor QQcs impact the removal efficiency of COD (>94%), phenol (>94%), and ammonium (>94%) indicating that QQ did not have adverse effect on treatment performance. Both BH4 and QQcs delayed the production of soluble microbial products and, thus, mitigated membrane fouling that delayed an increased transmembrane pressure. Interestingly, BH4 exhibited a higher filtration performance compared to QQcs, which might be attributed to the relatively higher degradation of short- and medium-chain N-acyl-homoserine lactones by BH4 and potential QQ microorganisms in QQcs [169]. In another study, a membrane bioreactor combined with the photo-Fenton process was applied to degrade phenol and other organic compounds. Coir retting wastewater was treated, in which the phenol concentration was 420 mg L^−1^. The treatment was carried out in an MBR for 1–15 h with an HRT of 8 h. The COD and phenol removals were determined to be 99% and 78%, respectively. The response surface methodology was utilized to optimize the photo-Fenton process [168]. Petrochemical effluent treatment poses a significant challenge for traditional anaerobic reactors from which gasification effluent has significant levels of phenol, cresols, and resorcinol. These phenolic chemicals are hazardous and inhibitory. Garcia et al. investigated the simultaneous decomposition of *p*-cresol, resorcinol, and phenol in anaerobic saline conditions. The saline condition (8 g Na^+^ L^−1^) was applied in two anaerobic membrane bioreactors, which were supplied with phenol-*p*-cresol or phenol–resorcinol mixtures. With influent concentrations of 1200 mg *p*-cresol L^−1^ and 2000 mg phenol L^−1^, the removal efficiency was nearly 100 percent. The complex solution of resorcinol and phenol at 800 mg resorcinol L^−1^ and 2000 mg phenol L^−1^ demonstrated a similar elimination efficiency. It was reported that the *Syntrophorhabdus* sp. and *Methanosaeta* sp. were the most common bacteria and methanogen in both the AnMBRs, respectively [170]. Another study demonstrated the treatment of coal gasification wastewater using an anoxic/aerobic MBR with and without PU foam carrier. The findings revealed that both systems effectively removed COD (>93%) and total phenols (>97%) but exhibited poor ammonia nitrogen removal (<35%) regarding the ammonia oxidation process. The microbial community demonstrated that *Flavobacterium* sp., *Holophaga* sp., and *Geobacter* sp. were abundant microorganisms found on PU foam [171]. 

## 8. Cellulose Membranes in MBRs and Wastewater Treatments

Cellulose membranes have drawn a lot of attention due to their distinctive features and advantages. Cellulose membranes are highly biodegradable as they are made from natural materials like wood pulp or cotton fibers, thus reducing the negative effects on the environment [195]. Their high affinity with water makes them hydrophilic, resulting in minimized membrane fouling. These characteristics prevent both organic and inorganic contaminants from sticking to the membranes’ surface [196]. Cellulose membrane surfaces can be modified by adding functional groups targeting specific contaminants’ removal or achieving preferential filtration based on molecular size, charge, and hydrophilic/hydrophobic properties [197,198]. Cellulose membranes are non-toxic and biocompatible, and they do not have any negative effects on the biological processes or microorganisms that are a part of the MBR system [199]. The application of cellulose membrane in MBRs encourages the development and attachment of microorganisms, facilitating the formation of biofilms, and improving treatment effectiveness. Compared with synthetic membranes, it requires less energy and lower costs. Their distinct structure and characteristic enable them high permeability, which lowers the energy required to maintain hydraulic pressure in the MBR system. Energy saving and operational cost reduction are important factors considered in MBR-based wastewater treatment. The application of cellulose membranes in MBRs adheres to the ideals of sustainable technology, encouraging environmental stewardship and lowering dependency on petroleum resources [200,201]. 

Current MBR membranes are made from synthetic polymers, including polysulfone (PS), polyvinylidene fluoride (PVDF), polyethersulfone (PES), and polyacrylonitrile (PAN) [202]. These membranes exhibit strong mechanical properties as well as chemical and thermal stability. Nevertheless, these synthetic polymeric membranes are susceptible to a decline in permeability caused by the build-up of solids, suspended particles, and other substances on the membrane surface and/or within the pores [203]. A recent study demonstrated that the nanostructured modification of cellulose by silica nanoparticles could serve to mitigate the membrane’s inclination toward filler aggregation. Consequently, this promoted the formation of a more uniformly consolidated structure within the nanostructured cellulose fibers, and, thus, a low-fouling cellulose membrane was successfully fabricated [204]. 

Another work reported a refined cellulose membrane having promising characteristics on wastewater collected from MBRs in a sewage treatment plant. The cellulose membrane was comprised of a lyocell microfiber scaffold infused with TEMPO-oxidized cellulose nanofibers (CNF) crosslinked using polyamideamine-epichlorohydrin (PAE). A high permeation flux of 127.6 ± 21.8 L m^−2^ h^−1^ bar^−1^, an exceptional separation efficiency exceeding 99.9%, a favorable flux recovery ratio surpassing 95%, and the capability of self-healing were achieved from cellulose membrane compared with commercial polymeric membranes, namely PVDF and PES [199]. TEMPO-modified cellulose nanofibers were reported to have improved the mechanical property and reduced the pore size of a membrane [198]. By incorporating cellulose nanofibers into the cellulose-based microfibers, the design effectively circumvented the common issue of delamination that often arises in membranes with layer-by-layer coatings. Apart from their anti-fouling properties, to improve the mechanical properties and to enhance the water flux and chemical resistance of cellulose membranes, cellulose nanofiber or nanocrystals can be easily blended with synthetic polymers such as polyamide [205] or other biopolymers such as chitosan [206] or alginate [207]. These biopolymer-blended membranes are excellent for a variety of MBR applications due to their enhanced mechanical strength, fouling resistance, and selectivity. This makes it possible to employ cellulose membranes in MBRs for wastewater treatment.

Recently, the filtration efficiency, anti-fouling performance, and flux recovery of nanofibrous composite cellulose nanofiber (TFNC-CNF)-coated membrane and PVDF membrane were compared. It was shown that the super-hydrophilic nature and negative charge on the surface of the TFNC-CNF membrane resulted in enhanced anti-fouling properties. The TFNC-CNF membrane had an initial flux recovery of 90% which was substantially higher than that of PVDF membrane, which showed a flux recovery of 26–43%, after mechanical cleaning. The permeability of the TFNC-CNF membrane ranged from 71.3 to 138.9 L m^−2^ h^−1^ bar^−1^ in the tests for continuous wastewater ultrafiltration. The analyzed variables demonstrated that TFNC-CNF membranes exhibited better potential as membranes for MBRs [208]. In another study, a solution casting phase inversion technique was applied to produce the amino-functionalized nanodiamond (ND) and polyethylene glycol (PEG)-grafted ND embedded cellulose acetate (CA) membranes. Properties such as the critical flux, fouling behavior, and anti-fouling properties against EPS formation of the pure and modified cellulose acetate membranes were studied in a bench-scale MBR system for filtering pharmaceutical wastewater. The results demonstrated that cellulose acetate membrane loaded with 0.5 wt% ND-PEG showed highest hydrophilic property as well as higher anti-fouling and greater critical flux. CA/ND-NH_2_ (0.5 wt.%) and CA/ND-PEG (0.5 wt.%) facilitated less formation of EPS that caused delay of bio-fouling and thus increase CA membrane durability. This was owing to the strong hydrogen bonding of the water molecule to the oxygen atoms in the PEG and a strong interaction between the amine group and water [209].

## 9. Techno-Economic Analysis of Wastewater Treatment Using Conventional and MBRs Technologies

MBRs are projected to be used more frequently in municipal and industrial wastewater treatment because of the high quality of their effluents. Steam stripping, activated carbon, pervaporation, and conventional activated sludge (CAS) are common technologies that compete with the MBRs for wastewater treatment. Most conventional processes produce solid or liquid waste streams e.g., sewage sludge, contaminated sorbent, or extractant, etc. that must be disposed of or further treated, resulting in additional expenses and treatment steps [210]. MBRs based wastewater treatment technologies employ a combination of biodegradation and micro/ultrafiltration and reverse osmosis methods, and therefore, treated water is advised for indirect potable reuse for various purposes e.g., agricultural, and industrial uses [18]. Although MBRs have advantages over the CAS method, the technology transition from CAS to MBR systems has been controversial owing to the excessive use of energy and membrane fouling in MBR applications. What about the cost-effectiveness of MBRs over CAS? It is critical to clarify these challenges by comparing the CAS ecosystem to that of MBRs [211,212]. The existing literatures have extensively analyzed the performance and cost of MBRs under various scenarios, including different temperatures, flux levels, aeration modes, membrane lifespans, and other relevant parameters [213]. The energy consumption, capital/operating costs (CAPEX/OPEX), and full life cycle costs of MBRs have been thoroughly evaluated [214,215] and compared with CAS processes [216].

As demonstrated in Table 3, the techno-economic analysis of conventional and MBR technologies for wastewater treatment consists of two major components: operational expenses (OPEX) and capital expenditures (CAPEX). Energy consumption as well as membrane maintenance and replacement are the main operational costs in MBRs, where energy consumption accounts for around 40–60% of the total operating costs [217]. The cost comparison on full-scale MBR wastewater reclamation system is scarce due to inadequate literature. Activated carbon appears to be more expensive than alternative technologies. Moreover, activated carbon systems do not adsorb dichloromethane effectively, and higher amount of carbon would be necessary for high organic concentration. Another membrane-based method for treating wastewater is the application of pervaporation system in which wastewater treatment takes place without microbial degradation [218]. 

In 2017, Iglesias et al. reviewed actual data on both CAPEX and OPEX for fourteen MBRs with capacities ranging from 288 to 35,000 m^3^/d. They compared hollow-fiber and immersed flat sheet membrane architectures and CAS system, and reported that the CAPEX of MBR was around 10% less than that of extended aeration with advanced reclamation treatment. The OPEX of MBR system was roughly 30% higher when an advanced water reclamation treatment was followed by a conventional water reclamation treatment. This OPEX analysis was carried out in treatment plants with capacities less than 10,000 m^3^/d. When compared with the prolonged aeration and advanced water reclamation treatment, the MBR treatment has superior cost conditions above this capacity and is 1.34 times more expensive [226]. In a study, Xiao et al. reported that the construction of wastewater treatment plants for biological treatment or MBR procedures accounted for 55–85% of the cost. The findings of this study, which was carried out in 2018, revealed that the CAPEX of MBRs for municipal wastewater treatment was around 600 USD/(m^3^/d), but the cost of MBRs for the treatment of industrial wastewater was approximately 900 USD/(m^3^/d) [222]. The CAPEX for the construction of a plant with a one megaliters per day flow capacity would be between USD 2.9 million and 6.9 million [220,221]. Because of the high input concentration and extended treatment time, MBR technology for industrial wastewater treatment often has higher CAPEX and a larger land footprint than that for municipal wastewater treatments. A cost analysis for the recycling of greywater using SMBR and a tubular nanofiltration (NF) process with two different production capacities of 3 and 30 m^3^/d was carried out by Humeau et al. Based on their report, the expenses associated with greywater recycling by a NF facility operating at a capacity of 3 m^3^/d were 7.80 EUR/m^3^. In contrast, on-site recycling using an SMBR incurred a cost of 7.40 USD/m^3^. When the plant’s capacity was expanded to 30 m^3^/d, the cost was nearly cut in half. Indeed, the direct expenses were 4.82 USD/m^3^ for the NF process and 4.40 USD/m^3^ for the SMBR [225]. The CAPEX of MBR construction for a wastewater treatment plant ranges from 6000 to 1000 USD/m^3^/d depending on the system’s productivity. The membrane plant itself, along with all the supporting equipment, accounts for 30–60% of the entire cost, which is a significant portion. The price range for membrane blocks is 75–150 USD/m^2^ with a typical specific productivity of 15–30 L/h/m^2^ of membrane area. The cost for treating household wastewater with hollow-fiber modules ranges from ~0.24 to 0.25 USD/m^3^ [224]. Another study by DeCarolis et al. found that the overall CAPEX and OPEX for 3785.4 m^3^/d capacity for MBR raw wastewater recycling systems were between 0.478 USD/m^3^ and 0.592 USD/m^3^. Based on the information presented, the overall expense of MBR facilities remains comparatively stable [223].

Although the OPEX of MBRs have been reported to be in a range two to four times higher than that of CAS, as shown in Table 3, indeed, assessing the overall technical and economic viability of MBRs solely based on costs is insufficient. High costs may be justified by corresponding high benefits. To provide a more comprehensive evaluation, it is crucial to consider net profit and technical efficiency, taking into account both inputs and outputs, which encompass costs and net profits [227]. A more reasonable measure of the techno-economic feasibility and effectiveness of MBRs involves a quantitative assessment of the environmental benefits associated with wastewater treatment. Based on CAPEX and OPEX data, the marginal cost and environmental benefits could be assessed (considering the shadow prices of pollutants), resulting in a net profit of approximately 35 CNY/m^3^ or roughly 4.9 USD/m^3^, on average, for MBR technology [228]. Therefore, a net profit is roughly evaluated as approximately 10 times greater than CAPEX+OPEX for MBRs reported in a range of 0.478 to 0.592 USD/m^3^ [223]. Nevertheless, the net profit and cost/energy efficiencies exhibited substantial variations based on factors such as geographical location, effluent standard, and operating year of the MBRs. A techno-economic model analysis additionally indicated that external factors like the regional economic level and population density also influenced the cost/energy efficiencies of the MBRs. To sum up, the economic viability of MBRs was particularly notable under strict effluent standards and in underground locations, besides there is a potential for improvement through technological advancements and policy incentives following environmental concerns.

## 10. Conclusions

The rigorous management of the chemical and biological substances in the effluents from petrochemical and pharmaceutical industries that are discharged into ground and surface water bodies is necessarily being implemented. Specific MBR configurations could retain, concentrate, and subsequently break down many of these chemicals without the requirement of any complex tertiary treatment procedures. It is possible to acclimate a large number of microorganisms and improve their reaction kinetics by retaining all biomass and biological catalysts inside the bioreactor to enhance biodegradation efficiency. MBR technology has significant potential in various applications such as solid waste digestion, odor control, and industrial wastewater treatment. The technical feasibility of this technology has already been proven through a series of bench-scale and pilot research. To remove important phenolic and aromatic contaminants from effluents, membrane-assisted hybrid systems are promising approach for significant advancement and attention regarding frontier development in membrane module fabrication in terms of more selectivity, longer durability, and lower cost. Future studies in this sector for sustainability, good health, and well-being will undoubtedly be sparked by the potential of combining the removal of organic matter, nutrients, hazardous substances, and biological organisms as well as downstream processing in a single and compact treatment system. In terms of techno-economic assessment, not only cost estimations but also the valuation of the associated environmental benefits of MBRs system are necessary to justify a suitable investment policy. Detailed cost–benefit analyses and technical efficiency evaluations of full-scale membrane bioreactors are crucial for a comprehensive understanding of their performance, economic viability, and sustainability.

## Figures and Tables

**Table 1:** Degradation of phenolic compounds by different microorganisms.

Type of Contaminants	Initial Concentration	Microorganisms	Contaminant Removal Efficiency (%)	Time	References
2,4-dichlorophenol	51 mg L^−1^	*Pseudomonas putida*	35	8 days	[82]
Phenol	1000 mg L^−1^	*Pseudomonas aeruginosa*, *Klebsiella pneumoniae*, *Klebsiella variicola*	71.70–74.67	3 days	[83]
Phenol	300 mg L^−1^	*Bacillus laterosporus BT-271*	100	15 days	[85]
Phenol	NA	*Rhodococcus erythropolis* (pSRKBphe-cat) *Rhodococcus erythropolis* (pSRKphe)	100 87	14 days	[86]
Phenol	2500 mg L^−1^	*Pseudomonas cepacia*	100	96 h	[87]
Phenol	1750 mg L^−1^	*Bacillus brevis*	100	132 h	[87]
Phenol	800 mg L^−1^	*Acinetobacter calcoaceticus*	91.6	48 h	[88]
Phenol	1.1 g L^−1^	*Acinetobacter lwoffii* NL1	0.5 g L^−1^	12 h	[89]
Bisphenol A	2.5 to 10.0 mg L^−1^	Consortia of immobilized microorganisms	87.1–92.9	NA	[90]
Phenol	1000 mg L^−1^	*Pseudomonas putida* (MTCC1194)	100	162 h	[91]
Catechol	500 mg L^−1^	*Pseudomonas putida* (MTCC1194)	100	92 h	[91]
Phenol	500 mg L^−1^	*Rhodococcus aetherivorans* UCMAc-603	35.7 mg L^−1^ h^−1^	NA	[92]
Phenol	1750 mg L^−1^	*Rhodococcus aetherivorans* UCMAc-603	18.2 mg L^−1^ h^−l^	NA	[92]
Phenol	NA	*Rhodococcus ruber* SD3	98	72 h	[93]
α-Naphthol	NA	*Oscillatoria rubescens*	59.49	5 days	[95]
Phenol and *p*-cresol	NA	*Pseudomonas putida* ATCC 17484	100	48 h	[96]
2,4-Dichlorophenol	NA	*Chlorella pyrenoidosa*	100	120 h	[97]
Bisphenol A	NA	*Desmodesmus* sp.WR1	18–57	10 days	[98]

Note: NA = not available.

**Table 2:** Comparison of phenol removal by different modified membrane bioreactors (MMBRs).

Membrane Bioreactor	Membrane Type	Contaminants	MBR Operating Parameters	Phenol Removal Efficiency	References
EMBR	PDMS/PMMA/MWCNTs	Phenol (1000–4000 mg L^−1^)	Saline wastewater; effective membrane surface area: 20 cm^2^; HRT: 24 h; and temperature: 24 ± 2 °C.	100%	[161]
EMBR	Hytrel™ 3548 tubing	Methyl ethyl ketone, benzene, phenol, and acetic acid (1000 mg L^−1^)	Synthetic hydraulic fracturing wastewater; T: 30 ± 0.5 °C; effective membrane surface area: 0.132 m^2^; V: 3 L; and HRT: ~8 h and microorganisms: microbial consortium (*Pseudomonas* sp., *Comamonas* sp., *Achromobacter* sp., *Lysinibacillus* sp., and *Oxalobacter* sp.).	Benzene and phenol: 99% Methyl ethyl ketone: 96% Acetic acid: 53%	[162]
EMBR	Electro-spun fiber of polydimethylsiloxane/polymethyl methacrylate	Synthetic phenol-laden saline wastewater (phenol: 14.1–290.7 mg L^−1^)	Effective membrane surface area: 0.0048 m^2^; HRT: 24 h; T: 24–26 °C; and microorganism: *ProtTeobacteria* and *Saccharibacteria.*	100%	[156]
EMBR	Silicone membrane (capillary membrane)	Benzene, toluene, ethylbenzene, and xylene (BTEX)	T: 25 °C; pH: 6.8–7.0; agitation rate: 300 rpm; and microorganism: *Pseudomonas putida* TX1 and BTE1.	75–99%	[163]
MBBR	Polypropylene and polyurethane	Phenol (0.1 g L^−1^) and ammonia (0.1 g L^−1^)	pH: 6.5; HRT: 2–12 h; and air flow rate: 2.15 L min^−1^; and microorganism: acclimatized bacterial consortium.	91.2%	[164]
MBBR	Polyethylene	Phenol (800 mg L^−1^) and saline (40 g L^−1^) wastewater	HRT: 18 h; T: 23 ± 2 °C; pH: neutral; dissolved oxygen (DO): 4–5 mg O_2_ L^−1^; and microorganism: activated biomass mixed consortia.	99%	[165]
MBBR	Polyethylene	Synthetic wastewater; phenol (200 mg L^−1^)	HRT: 24 h; DO: 5.0 ± 1.0 mg L^−1^; pH: 7.0; T: 32 ± 2.0 °C; and microorganism: *Bacillus cereus.*	87.64%	[166]
MBR	Flat sheet membrane from methacrylate	Hypersaline wastewater, phenol (8–15 mg L^−1^) and salt (150–160 mS cm^−1^)	HRT: 0.5–0.7 days; and pH: 7.5–8.3; and microorganism: *Halomonas* and *Marinobacter.*	>98%	[167]
MBR	Flat sheet PVDF membrane	Coir retting wastewater; phenol (90 mg L^−1^)	HRT: 8 h; pH: 6.5-7.2; effective membrane surface area: 0.8 m^2^; and microorganism: activated sludge.	99%	[168]
MBR	Flat sheet ceramic membrane	Phenol-rich pharmaceutical wastewater	Phenol: 539 ± 67 mg L^−1^; HRT: 18 h; T: 27 ± 1 °C; pH: pH: 8.0 ± 0.5; effective membrane surface area: 0.008 m^2^; and microorganism: *Rhodococcus* sp.	>99%	[169]
AnMBR	PVDF membrane	Synthetic wastewater *p*-cresol (1200 mg *p*-cresol L^−1^) and phenol (2000 mg phenol L^−1^), resorcinol (800 mg resorcinol L^−1^) and phenol (2000 mg phenol L^−1^)	HRT: 6 d; T: 35 °C; operation time 77–112 d; and microorganism: *Syntrophorhabdus* sp. and *Methanosaeta* sp.;	100% 100%	[170]
A/O-MBR	PVDF hollow-fiber membrane	Coal gasification wastewater with abundant phenols	HRT: 12 h and 47 h; T: 20–25 °C; total membrane surface area: 0.2 m^2^; and microorganisms: *Flavobacterium* sp., *Holophaga* sp., and *Geobacter* sp.	>97%	[171]
SMBR	Polyvinylidene fluoride	Synthetic phenolic wastewater (phenol: 1000 mg L^−1^)	HRT: 16.6 h; T: 23–24 °C; effective membrane surface area: 5 × 10^−2^ m^2^; DO: 2 mg L^−1^; and microorganisms: activated sludge.	>99%	[154]
HFMBR	Trioctylphosphine oxide (TOPO) impregnated in polypropylene	Phenolic wastewater (phenol: 100 mg L^−1^)	HRT: 12 h; pH: 6.5–7.0; and microorganisms: *Pseudomonas putida ATCC 11172.*	100%	[172]
HFMBR	Polyether sulfone + granular activated carbon	Synthetic wastewater (phenol: 1000 mg L^−1^)	HRT: 18 h; and microorganisms: *Pseudomonas putida.*	100%	[173]

Note: EMBR: extractive membrane bioreactor; MBBR: moving bed biofilm reactor; MBR: membrane bioreactor; AnMBR: anaerobic membrane bioreactor; A/O MBR: anoxic/aerobic membrane bioreactor; SMBR: submerged membrane bioreactor; and HFMBR; hollow-fiber membrane bioreactor.

**Table 3:** Capital, operational, and unit wastewater treatment cost of conventional and MBR technologies.

Wastewater Treatment Technologies	Wastewater Type	Capital/Operational Cost (CAPEX/OPEX)	References
CAS	Municipal wastewater	0.11 USD/m^3^	[217,219]
Pervaporation	Organic-contaminated Wastewater	Capital cost: USD 180K Operating cost: USD 50K	[218]
MBR	Wastewater	Capital cost USD 2.9–6.9 million (1 megaliter per day flow capacity plant)	[220,221]
MBR	Municipal wastewater Industrial wastewater	Capital cost: 600 USD/(m^3^/d) Capital cost: 900 USD/(m^3^/d)	[222]
MBR	Municipal wastewater	0.48–0.59 USD/m^3^	[223]
HFMBR	Municipal wastewater	0.55–0.68 USD/m^3^	[213]
HFMBR	Domestic wastewater	0.24–0.25 USD/m^3^	[224]
SMBR	Greywater (operating capacity 3 m^3^/day) (operating capacity 30 m^3^/day)	SMBR: 7.40 USD/m^3^ NF: 7.80 USD/m^3^ SMBR: 4.40 USD/m^3^ NF: 4.82 USD/m^3^	[225]
FSMBR	Municipal wastewater	0.42 USD/m^3^	[213]

Note: CAS: conventional activated sludge; MBR: membrane bioreactor; SMBR: submerged membrane bioreactor; HFBMR: hollow-fiber membrane bioreactor; and FSMBR: flat sheet membrane bioreactor (1 EUR is approximately 1 USD in January 2024).
